# Cognitive Relevance Transform for Population Re-Targeting

**DOI:** 10.3390/s20174668

**Published:** 2020-08-19

**Authors:** Gregor Koporec, Andrej Košir, Aleš Leonardis, Janez Perš

**Affiliations:** 1 Gorenje, d. o. o., Partizanska cesta 12, SI-3320 Velenje, Slovenia; 2 Faculty of Electrical Engineering, University of Ljubljana, Tržaška cesta 25, SI-1000 Ljubljana, Slovenia; andrej.kosir@fe.uni-lj.si; 3 School of Computer Science, University of Birmingham, Edgbaston, Birmingham B15 2TT, UK; a.leonardis@cs.bham.ac.uk

**Keywords:** cognitive relevance, deep learning, crowd-sourcing, target user population, categorization, classification

## Abstract

This work examines the differences between a human and a machine in object recognition tasks. The machine is useful as much as the output classification labels are correct and match the dataset-provided labels. However, very often a discrepancy occurs because the dataset label is different than the one expected by a human. To correct this, the concept of the target user population is introduced. The paper presents a complete methodology for either adapting the output of a pre-trained, state-of-the-art object classification algorithm to the target population or inferring a proper, user-friendly categorization from the target population. The process is called ‘user population re-targeting’. The methodology includes a set of specially designed population tests, which provide crucial data about the categorization that the target population prefers. The transformation between the dataset-bound categorization and the new, population-specific categorization is called the ‘Cognitive Relevance Transform’. The results of the experiments on the well-known datasets have shown that the target population preferred such a transformed categorization by a large margin, that the performance of human observers is probably better than previously thought, and that the outcome of re-targeting may be difficult to predict without actual tests on the target population.

## 1. Introduction

**Figure 1:**
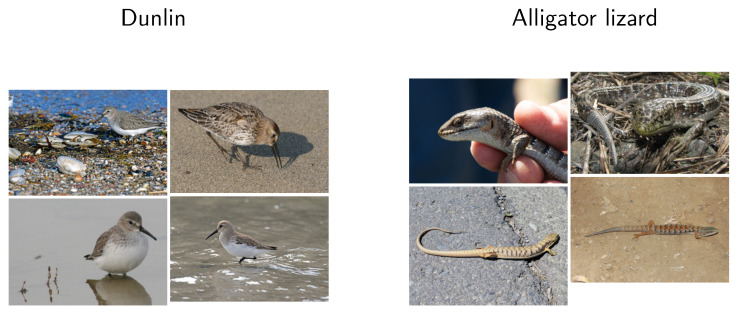
Samples of <dunlin> and <alligator lizard>. By the affordances <dunlin> is different from <alligator lizard> as they are different species. By the visual information, they have similar color and texture. The experiments showed humans will label images into semantically different categories, but AlexNet CNN, trained on ImageNet dataset [11], would misclassify <dunlin> as <alligator lizard>. <alligator lizard> photos by Jerry Kirkhart, California Department of Fish and Wildlife, and Greg Schechter licensed under CC BY 2.0 and by Eugene Zelenko licensed under CC BY-SA 2.0.

Humans have a different perception of categories than Convolutional Neural Networks (CNNs). For one, CNNs use exclusively visual features to perform the classification. Aside from the low-level visual features, CNNs also take advantage of the spatial structure of these features, i.e., how these features are located [1]. However, humans think differently and explore different, high-level features on images to perform a classification task [2,3,4,5,6,7,8]. Thus, for a human, the images of a bird <dunlin> and a reptile <alligator lizard> (Figure 1) fall into different categories, as humans take into the account the high-level concept of *affordances*—perceivable action possibilities (i.e., only actions that depend on users’ physical capabilities, their goals and past experiences) [9]. The algorithm, however, focuses only on visual information (color, shape, and texture) [10]. Because both types of animals are similar in color and have a similar texture (see Figure 1), they fall into the same category despite being semantically different. From the human perception point of view, such a categorization is wrong and should be punished more severely (with greater penalty) in the training or evaluation process.

In the context of a CNN as a human assistant, the question is which pre-trained object classification algorithm better imitate target-users? The answer is the one that will separate semantically different images as much as possible, but how can such an algorithm be found? Images from the evaluation set could be labeled by human subjects and then the answers and algorithm results could be compared. However, the new problem immediately arises as people label the same image differently. Following the theory of cognitive relevance [12], the labels are based on the context and processing effort which is a matter of human experience and cultural background. In view of the unchanged context, people will be able to label the image with *synonyms* (words that denote the same concept and are interchangeable in many contexts), *hypernyms* (words that denote the more broad meaning of a concept), and *hyponyms* (words that denote a more specific concept). Considering the hierarchical structure of the lexical database, different human labels are positioned vertically. Following the concept of cognitive relevance [12], it may happen that people change the context and label the image completely differently. In this case, they move horizontally across the hierarchical structure.

A large population can be used to solve the problem of image multi-labeling. Horizontal movements in the lexical database can be considered outliers and are removed with the appropriate analysis. After that, the vertical point in the lexical hierarchy can be defined, which is consistent with the principle of maximizing relevance [12]. The located point in the lexical hierarchy is a lexical representation of a concept to which the target population gets the association when it sees a specific image. However, a lexical representation of the image in a categorization dataset, used for the evaluation of deep learning algorithms, can be different. For example, approximately half of 1000 ILSVRC2012 (ImageNet Large Scale Visual Recognition Challenge 2012) object detection dataset categories are so-called ‘leaf’ categories, i.e., they represent very fine narrow concepts (e.g., breeds of dogs, types of mushrooms, exotic species of birds). A general population that would naturally prefer such categorization is probably very difficult to find. Using such a dataset to evaluate human classification performance is not appropriate.

CNNs are often tested on problems that contain hundreds of categories. ILSVRC2012’s 1000 categories are not, by definition, an open-world problem (all possible answers are specified). In practice, the 1000 category dataset-provided labels that cannot be effectively parsed by humans. For humans, a 1000 class classification is effectively an open-world problem. Therefore, people may provide a *good answer* which is not *correct*. It does not correspond to the label, as defined by the dataset creators. However, an evaluation makes sense only if the categories of the dataset are transformed into the human perception of the target population (*user population re-targeting*).

Essentially, by adapting the dataset for the target user population the bias is introduced into the dataset. Contrary to the biases already contained in the categorization datasets [13,14,15,16,17] that negatively affect the evaluation of algorithms, this kind of bias helps in comparing humans to machines and finding the algorithm that resembles human cognitive processes as closely as possible. To compare humans to machines and find out which images cannot be distinguished by the algorithms, it makes sense to also adapt the algorithms’ responses vertically in the lexical hierarchy.

### Scope of the Research

There are differing views on *categorization* in cognitive science [18]. The view, related to the concept of affordances and described above, is considered to be *the modern view*. In contrast, *the classical view* of the category relies on the concept of *shared properties* instead of affordances. For example, both sparrows and penguins have wings and thus they are birds in a biological sense, even though penguins cannot fly.

There is a good reason why a modern view on categorization (based on *prototype theory* and related to affordances) has gained prominence—it has been found that categorization in people is complex beyond what could be explained by shared properties [18]. This does not mean that the classical view itself is wrong, but its applicability is limited. This research focuses on *substituting a human helper* with *an AI-powered machine* and therefore, a modern view on categorization has to be taken into account.

There are, of course, many use cases for AI-powered classification, where the classical view of the categorization (also called *objectivist* view [18]) is more appropriate, and the categories have to be rigidly defined by an expert. However, the scope of this research is limited to the instances where human reasoning regarding the categorization is imitated, which brings unique challenges, addressed in this work.

**Figure 2:**
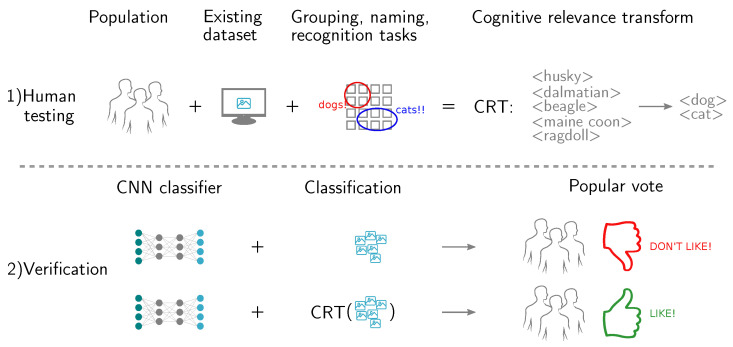
Main idea of this work. Images from the existing dataset are shown to the target population. Members of a population perform grouping, category naming, and recognition tasks on a subset of images. The results are used to find a Cognitive Relevance Transform (CRT) that modifies the number, grouping, and naming of categories. The CRT is population-specific and *introduces bias* into the transformed dataset. As a result, even a disjointed group of people, sampled from the same population, prefers the classification results on the transformed dataset, compared to the results on the original dataset.

In this work, a new user study-based approach to transform existing datasets into the datasets that are tailored to the specific user population is introduced. This approach has two benefits: it can reevaluate both algorithm and human performance, and increase user satisfaction with the output of the classification algorithm. As shown in Figure 2, images from the selected dataset are shown to the target user population. Members of a population perform grouping, category naming, and recognition tasks on a subset of images. The results are used to find a *Cognitive Relevance Transform* (CRT) that modifies the number, grouping, and naming of categories. By *user population re-targeting* with the CRT dataset-provided labels, CNNs’ outputs, and human-provided labels are then modified to be able to compare humans to machines.

The developed methodology is generic to the point that can be used for existing datasets and algorithms to properly compare humans to algorithms. The CRT can be employed by anyone seeking to develop a user-friendly AI algorithm or to find one among existing ones. It can also be used for future datasets to determine more relevant categories. Note that the proposed methodology specifically addresses the use of classification methods in situations where the end user of a classification system is a human. In many applications this is not the case (e.g., autonomous driving or industrial applications), but with AI-powered appliances having ever greater roles in human lives and decisions, this aspect of classification will only increase in importance.

The paper is organized as follows: After the related work (Section 2), a *Cognitive Relevance Transform* that transforms the structure of the dataset is defined in Section 3. How to utilize user population re-targeting is demonstrated in Section 4. In the experiments (Section 5), the materials and methodology for conducting similar experiments on ILSVRC2012 and VireoFood-172 datasets are described. The results and discussion for the ILSVRC2012 dataset are presented in Section 6. Section 7 presents the results and discussion for the VireoFood-172 dataset. This paper concludes with Section 8.

## 2. Related Work

After CNNs outperformed other classifiers on the ILSVRC2010 challenge [19], deep learning has become a de facto methodology [20]. Still, CNNs are not perfect and many challenges still need to be solved [21]. One of the challenges is human’s trust in their answers. As researchers demonstrated, there exist colored patterns that are unrecognizable to humans, but CNNs recognize familiar objects with ≥99.6% certainty [22]. Others have shown that the so-called adversarial examples—images with perturbations, not visible to human eye—can also fool the CNNs [23]. Previous studies [22,24,25] pointed out that the problem arises due to adversarial examples occupying a much larger area in input space than training examples.

The literature did not provide any research that considers *user population re-targeting* as an approach to get closer to human classification. Most of the work only considers the comparison of humans to a machine classification performance. The majority is focused on low-level recognition performance studies, based on visual distortions of images or the viewpoint variations of objects. Dodge and Karam [26,27] studied the performance with Gaussian blur and additive Gaussian noise. Both works neglected the fact that the high distortions could be irrelevant to humans with higher cognitive recognition capabilities, similarly to Wichmann et al. [28] (contrast reduction). The results have indicated that human observers and CNNs exhibited a similar performance, but the humans were more robust to contrast changes. The viewpoint variation comparisons between humans and CNNs were done by Kheradpisheh et al. [29,30]. The humans and the CNNs were correlated in the viewpoint variation comparisons, but the error distributions of computational models were different from the humans.

In contrast, Stabinger et al. [31] studied higher-level abstraction capabilities of CNNs and human subjects. The CNNs were trained and tested on the SVRT framework presented by Fleuret et al. [2]. The framework consisted of a series of 23 classification problems with randomly generated shapes. The shapes were unknown to humans, and so the prior knowledge of humans to solving problems was minimized. The classification problems were relationships between the shapes on images. Stabinger et al. [31] reported that CNNs were generally not capable of solving problems containing shape comparison.

Abstraction experiments could be used for higher cognitive capabilities, but researchers Linsley et al. [8], Pramod and Arun [4] showed that humans and algorithms had distinct strategies to solve the problems. Linsley et al. [8] identified visual features used by humans and CNNs during object recognition. The visual features were represented as importance maps for individual images. The authors observed that the maps were strongly stereotyped across the subjects and the CNNs favored different visual features. Pramod and Arun [4] used a different approach with perceived dissimilarity measurements. They collected a large dataset of perceived dissimilarity measurements and used it to train and test computational models. They perceived that the dissimilarity estimation for human subjects was defined as the reciprocal of the average search time of the target among multiple identical distracting objects. The authors reported the existence of systematic differences between the object representations in humans and machines.

The discovery of systematic differences between humans and machines contributed to the development of bio-inspired computational models that would overcome the performance gap. It is believed that a human IT cortex is mainly responsible for the higher cognitive performance in recognition tasks. By Yamins et al. [32] it would thus be necessary for computational models to strongly correlate to the IT. Yamins et al. [32] showed that hierarchical neural networks (HMO models) were highly predictive of the IT cortex. By testing the models on recognizing photorealistic 3D models with different position, scale, and pose, the models matched the human performance. Similarly, Cadieu et al. [33] compared the IT cortex responses to CNNs on recognizing objects with viewpoint variations. They also found that CNNs achieved equal performance to the IT cortex and that they were even better than the bio-inspired HMO models. Still, both works tested only low-level cognitive capabilities. A more high-level study was done by Rajalingham et al. [34]. They observed the object recognition behavior of humans, monkeys, and CNNs on the object-level and image-level discriminability. Rajalingham et al. [34] showed that the CNNs accurately predicted primate patterns of how often the object was incorrectly categorized, but the discriminability of each image from all other objects was significantly different.

All related research shows that regardless of the type of an experiment, there exists a gap between computational models and humans. However, there is no simple approach or metric to evaluate the models, according to their human-like capabilities. Therefore, in this work, it is demonstrated how to use a simple tool (Cognitive Relevance Transform) to truly compare models to humans to get closer to human classification. This tool can be used to normalize already established metrics in object classification to human cognitive relevance capabilities. Transformed metrics are comparable to humans and can clearly show which model has more human-like properties.

## 3. The Cognitive Relevance Transform

The Cognitive Relevance Transform (CRT) is defined as a sequence of operations on image categories, with each operation belonging to one of the three operation classes: (1) Merging of the multiple dataset categories into a new category, denoted by *E*; (2) Separating the dataset category into multiple categories, denoted by *S*; and (3) Renaming the category, denoted by *R*.

The CRT is obtained in three steps (see Algorithm 1). Intention is to assess the cognitive relevance of the whole dataset D⊂H×C where H is a set of images and C is a set of categories. A modern object detection/recognition dataset contains a huge number of both categories C and images H. The cost of testing humans on such a dataset would be prohibitive. So, a representative subset $D^{\prime }\subset H^{\prime }\times C^{\prime }$, using fewer categories and images per category, needs to be extracted. In the second step, a battery of tests (experiment tasks) on the human population *P* using subset $D^{\prime }$ is performed to get image groups as feature vectors *X* and labels *A*. This work presents the methodology on how to do this properly, to reduce the human workload, but still get useful results. In the third step, the CRT is derived from analyzing the grouping and labeling tasks in user studies.

**Algorithm 1** The Cognitive Relevance Transform.The Cognitive Relevance Transform (CRT) is a sequence of operations (E,S,R) in image categories. The CRT is obtained in three steps. First (operation 1), the dataset is subsampled by Algorithm 2. Second (operation 2), user studies are performed (see Section 3.2 and Algorithm 3). Third (operation 3), merging operations *E* and separation operations *S* are determined by Algorithm 4. Renaming operations *R* are determined by Algorithm 5. = denotes definition and ← denotes calculation.
**Require:**
D⊂H×C,P
**Ensure:** CRT 1: $D^{\prime }\leftarrow$dataset subsampling(*D*) 2: (X,A)←user studies($P,D^{\prime }$) 3: CRT=(E,S,R)←get crt($X,A,D^{\prime }$) 4: **function**
get crt($X,A,D^{\prime }$) 5:   (E,S)←determining merging and separation operations($X,D^{\prime }$) 6:   R←determining renaming operations(*A*) 7:   **return**
(E,S,R) 8: **end function**

### 3.1. Reduction of the Dataset Size

Reduction of the dataset size can be done by Algorithm 2, which requires the total number of categories |C|, a maximal number of categories per task $\nu_{c}$, a number of images per category $\nu_{i}$, categories’ effect size $h_{c}$, images’ effect size $h_{i}$, significance level α, and statistical power 1−β. The algorithm ensures the minimal number of categories $\left|C^{\prime }\right|$, the minimal number of images per category $\left|H^{\prime }\right|$, and the number of batches |B| that are needed to evaluate all images from a subset $D^{\prime }$. Batch *B* is a set of categories and images $B\subset D^{\prime }$ that can fit into one task.

**Algorithm 2** Dataset Subsampling.To reduce the dataset size, the minimum number of categories $n_{c}$ is calculated by the statistical power of two-sample test for proportions with unequal sample sizes. For details, see [35]. The minimum number of images per category $n_{i}$ is calculated by the statistical power of $\chi^{2}$ statistical test of independence [35]. Both, $n_{c}$ and $n_{i}$ are then corrected to a discrete number by operation 6. Finally, the number of batches |B| is calculated. ← denotes calculation.
**Require:**
$\left|C\right|,\nu_{c},\nu_{i},h_{c},h_{i},\alpha ,1-\beta$

**Ensure:**
$\left|C^{\prime }\right|,\left|H^{\prime }\right|,\left|B\right|$
 1:$n_{c}\leftarrow$pwr.2p2n.test($\left|C\right|,h_{c},\alpha ,1-\beta$) 2: $n_{i}\leftarrow$pwr.chisq.test(${\left(n_{c}-1\right)}^{2},h_{i},\alpha ,1-\beta$)$/n_{c}$ 3: $|C^{\prime }|\leftarrow$to integer($n_{c},\nu_{c}$) 4: $|H^{\prime }|\leftarrow$to integer($n_{i},\nu_{i}$) 5: $\left|B\right|\leftarrow \left(\left|C^{\prime }\right|\left|H^{\prime }\right|\right)/\left(\nu_{c}\nu_{i}\right)$ 6: **function**to integer(n,ν) 7:   **return**
$\lceil \frac{n}{\nu }\rceil \nu$ 8: **end function**

The minimum number of categories $n_{c}$ is determined by the *statistical power of two-sample test for proportions with unequal sample sizes* (operation 1 in Algorithm 2). The null hypothesis for the operation is *no difference in the size proportion of the original dataset and its subset*. $n_{c}$ is then corrected to final count $\left|C^{\prime }\right|$ by function TO INTEGER in Algorithm 2 operation 6. With this operation, $\left|C^{\prime }\right|$ is established as: (1) a discrete number; and (2) divisible by $\nu_{c}$. The first condition ensures whole categories. The second condition ensures the categories can be equally divided among tasks in user studies.

The minimum number of images $n_{i}$ is determined by the *statistical power of $\chi^{2}$ statistical test of independence* (operation 2 in Algorithm 2), where the null hypothesis is *two random variables are independent*. The reason behind using such a test is that one of the results after the human experiment will be a human confusion matrix for the selected categories. The confusion matrix consists of actual and predicted class variables. The basic idea is that a predicted class would mainly indicate the same actual class (they are dependent). Then, $n_{i}$ is corrected to final count $\left|H^{\prime }\right|$ by function 6 in Algorithm 2 to ensure the same conditions described for $\left|C^{\prime }\right|$.

The number of batches |B| is calculated by operation 5 in Algorithm 2. Because the categories and images can be equally divided among tasks (ensured by function 6 in Algorithm 2), all batches have equal size.

### 3.2. User Studies

To get the most out of user studies, the proper size of human population *P* must be first determined by Algorithm 3. The algorithm requires batch size |B|, minimum probability of successful classification by humans $p_{m}$, the estimated probability of successful classification by humans $p_{e}$, and statistical power 1−β. It ensures the total number of human subjects |P| and an estimation of a number of observations per image $n_{o}$.

**Algorithm 3** Population size.Effect size $h_{o}$ for the number of observations $n_{o}$ is calculated by operation 1 (see [35]). Next, $n_{o}$ is determined by the statistical power of the binomial test [35]. Finally, the number of batches |B| and the number of human subjects |P| are calculated. ← denotes calculation.
**Require:**
$\left|B\right|,p_{m},p_{e},1-\beta$

**Ensure:**
$\left|P\right|,n_{o}$
 1: $h_{o}\leftarrow 2\arcsin\left(\sqrt{p_{m}}\right)-2\arcsin\left(\sqrt{p_{e}}\right)$ 2: $n_{o}\leftarrow$pwr.p.test($h_{o},1-\beta$) 3: $\left|P\right|\leftarrow \left|B\right|n_{o}$

Estimation of a number of observations per image $n_{o}$ is done by *the statistical power of the binomial test* (operation 2 in Algorithm 3) with the effect size $h_{o}$. $n_{o}$ significantly determines a change in the image’s category as each image is checked if there exists a difference between a dataset-provided and a human-labeled category.

The total number of human subjects |P| for the whole experiment is determined by operation 3 in Algorithm 3, as each batch *B* can fit in one task and all images in *B* must be evaluated $n_{o}$ times.

**Figure 3:**
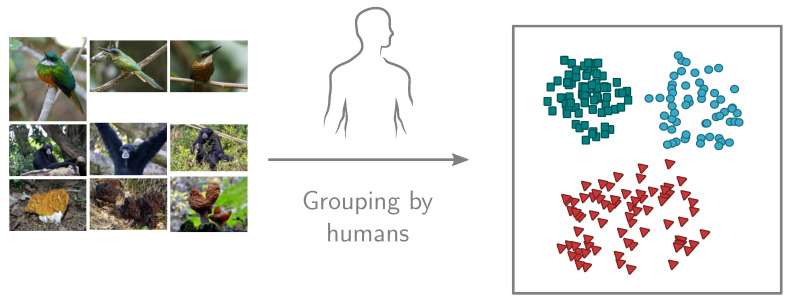
The participants are asked to group similar images as they see fit in Task 1. <jacamar> photos by Bernard Dupont licensed under CC BY-SA 2.0 and by David Surtees licensed under CC BY 2.0. <siamang> photos by cuatrok77 licensed under CC BY-SA 2.0 and by Abi Skipp licensed under CC BY 2.0. <gyromitra> photos by Tatiana Bulyonkova licensed under CC BY-SA 2.0 and by Andrey Gaverdovsky licensed under CC BY 2.0.

The experiment on the human population *P* is divided into two tasks, with clearly defined goals. A sequence of tasks guarantees that the information is revealed to the participants incrementally, so no cross-contamination occurs between the tasks. In the first task, the participants are asked to group similar images as they see fit (Figure 3). The goal is to evaluate which categories from the dataset $D^{\prime }$ are perceived as one category and which are separate categories by the human population *P*. The number of categories $\nu_{c}$ and the number of images in the category $\nu_{i}$ are unknown to the participants.

**Figure 4:**
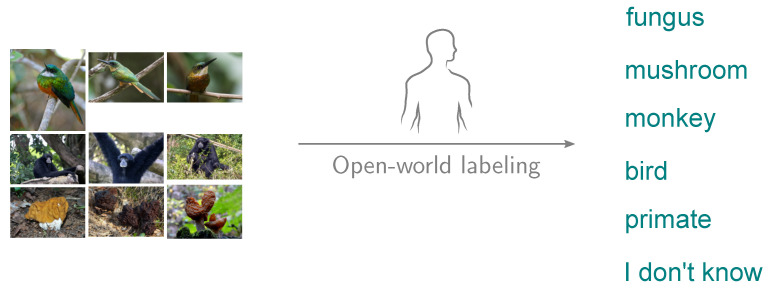
The participants in Task 2 label the images as they see fit. <jacamar> photos by Bernard Dupont licensed under CC BY-SA 2.0 and by David Surtees licensed under CC BY 2.0. <siamang> photos by cuatrok77 licensed under CC BY-SA 2.0 and by Abi Skipp licensed under CC BY 2.0. <gyromitra> photos by Tatiana Bulyonkova licensed under CC BY-SA 2.0 and by Andrey Gaverdovsky licensed under CC BY 2.0.

In the second task, the participants are shown the next sequence of images. They are asked to label the images as they see fit (Figure 4). The goal of this task is to determine the renaming operation.

### 3.3. Deriving CRT operations

The grouping task gives a set of feature vectors $X={\left\{x_{j}\right\}}_{j\leq |D^{\prime }|}$ where every feature vector $x_{j}={\left\{\nu_{h}\right\}}_{h\leq |P|}$ represents an image from a subset $D^{\prime }$ and its attributes $\nu_{h}$ represent group identification number (ID) from each human grouping. Group IDs are unique in such a way that two groups created by different human subjects have different IDs despite being created from the same batch of images *B*. Set *X* is then used in Algorithm 4.

**Algorithm 4** Merging and Separation operations.To determine merging operations *E* and separation operations *S*, a set of feature vectors *X* is clustered into a set of *C* clusters. Then, for each cluster $C_{i}$ and category $C_{j}^{\prime }$ get $n_{j}^{i}$ as a relative frequency of $C_{j}^{\prime }$ clustered into $C_{i}$. Use $n_{j}^{i}$ to separate the category $C_{j}^{\prime }$. In context of a cluster $C_{i}$, separation of $C_{j}^{\prime }$ is denoted by $s_{j}^{i}$. A set of separation operations for all categories $C^{\prime }$ in the context of a cluster $C_{i}$ is denoted by $S_{i}$. Merging operation $E_{i}$ in the context of cluster $C_{i}$ is calculated by merging results from separation operations $S_{i}$. = denotes definition and ← denotes calculation.
**Require:**
$X={\left\{x_{j}\right\}}_{j\leq |D^{\prime }|},x_{j}={\left\{\nu_{h}\right\}}_{h\leq |P|},D^{\prime }\subset C^{\prime }\times H^{\prime }$

**Ensure:**
E,S
 1: C←clustering(*X*) 2: (E,S)←get crt operations($C,D^{\prime }$) 3: **function**
get crt operations($C,D^{\prime }$) 4:   **for**
i≤|C|
**do** 5:    **for**
$j\leq |C^{\prime }|$
**do** 6:     $n_{j}^{i}\leftarrow$frequency($C_{j}^{\prime },C_{i}$) $/|H^{\prime }|$ 7:     $s_{j}^{i}=$separate($C_{j}^{\prime },n_{j}^{i}$) 8:    **end for** 9:    $S_{i}={\left\{s_{j}^{i}\right\}}_{j\leq |C^{\prime }|}^{i}$ 10:    $E_{i}=$merge($S_{i}$) 11:   **end for** 12:   **return**
$\left(E={\left\{E_{i}\right\}}_{i\leq |C|},S={\left\{S_{i}\right\}}_{i\leq |C|}\right)$ 13: **end function**

The clustering operation 1 in Algorithm 4 returns $C={\left\{C_{i}\right\}}_{i\leq k}$ clusters where each cluster $C_{i}$ contains images that the majority of human subjects agree to belong together. Clusters *C* are then used to determine the operations to transform the original categories to the newly created clusters by operation 3 in Algorithm 4.

For each cluster $C_{i}$ a set of separation operations $S_{i}={\left\{s_{j}^{i}\right\}}_{j\leq |C^{\prime }|}^{i}$ and a merging operation $E_{i}$ are proposed. Each separation operation $s_{j}^{i}$ defines a separation of category $C_{j}^{\prime }$ in the context of a cluster $C_{i}$. Separation operation $s_{j}^{i}$ is calculated using the relative frequency $n_{j}^{i}$ (see operation 6 in Algorithm 4), which is the percentage of images from category $C_{j}^{\prime }$ that can be assigned to cluster $C_{i}$. Merging operation $E_{i}$ in the context of cluster $C_{i}$ is calculated by merging results from separation operations $S_{i}$. In other words, merging operation $E_{i}$ combines images that can be assigned to cluster $C_{i}$ regardless to which category they originally belong.

Clusters *C* can be described by the most frequent label derived from Algorithm 5. A set of labels *A* from the second task is cleaned (denoted by $A^{*}$) and structured into a set of feature vectors *Y*, where every feature vector $y_{j}$ represents an image from a subset $D^{\prime }$ and its attributes $a_{k}^{*}$ represent a cleaned label from each human labeling. *Y* is then clustered in the same way as *X* because they are dependent. Each cluster $C_{i}$ is then assigned the most frequent label from the corresponding label cluster $Z_{i}$.

**Algorithm 5** Determining Renaming operations.A set of labels *A* is cleaned by the number of text operations. Cleaned labels $A^{*}$ are structured into a set of feature vectors *Y* and clustered into a set of *Z* clusters. Renaming operation $R_{i}$ for cluster $Z_{i}$ is determined by the most frequent label. = denotes definition and ← denotes calculation.
**Require:**
$A={\left\{a_{h}^{j}\right\}}_{h\leq |P|}^{j\leq |D^{\prime }|}$

**Ensure:**
*R*
 1: $A^{*}\leftarrow$clean(*A*) 2: $Y={\left\{y_{j}\right\}}_{j\leq |D^{\prime }|},y_{j}={\left\{a_{h}^{*}\right\}}_{h\leq |P|}$ 3: Z←clustering(*Y*) 4: $R={\left\{R_{i}\right\}}_{i\leq |Z|}\leftarrow$most frequent label(*Z*) 5: **function**
clean(*A*) 6:   $A^{*}\leftarrow$to lowercase(*A*) 7:   $A^{*}\leftarrow$decode to closest ASCII code($A^{*}$) 8:   $A^{*}\leftarrow$remove non-alphabetic characters($A^{*}$) 9:   $A^{*}\leftarrow$abbreviations to whole words($A^{*}$) 10:   $A^{*}\leftarrow$strip white space($A^{*}$) 11:   $A^{*}\leftarrow$word segmentation($A^{*}$) 12:   $A^{*}\leftarrow$tokenize words($A^{*}$) 13:   $A^{*}\leftarrow$keep nouns, adjectives and prepositions($A^{*}$) 14:   $A^{*}\leftarrow$spell check($A^{*}$) 15:   $A^{*}\leftarrow$lemmatize($A^{*}$) 16:   **return**
$A^{*}$ 17: **end function**

## 4. User Population Re-Targeting

The task is to compare and adapt machine algorithms to the human population *P*. First, machine classification is done on a subset $D^{\prime }$. For images in $H^{\prime }$, the algorithm normally outputs a score or a confidence value for categories in $C^{\prime }$. The algorithm’s top guess is then considered as the correct answer (Top-1 classification). Second, $n_{o}$ unique human subjects categorize each image in $H^{\prime }$. They can only choose one category from $C^{\prime }$ for each image.

Given both the output of the human and machine classification on a subset $D^{\prime }$, classifications can be evaluated by comparing confusion matrices as they are the most information-rich representations of classifier performance. However, there exist fundamental differences: the machine algorithm provides one result (albeit it can, in some cases, provide the confidence scores for multiple hypotheses), but the human population will provide one answer per human subject. The comparison between a population and a machine is therefore not entirely straightforward, as subjects differ in their knowledge (and possibly attention).

To address this problem, the individual answers by human subjects are essentially transformed into the population answers by Algorithm 6. The initial assumption is that original labels provided by the dataset creators $L^{\prime }$ are correct (but of course, not optimal), as the dataset was built using human annotators. Answers by human subjects are considered *a two-class problem*. Each image can be classified as a positive class (most frequent label) or a negative class (all the other labels). The minimum probability $p_{e}$ is then required for human subject consensus to use the positive class label ${\hat{l}}_{j}$ as the consensus label $l_{j}^{*}$ instead of the original label $l_{j}^{\prime }$. As shown in experiments, this results in very small changes to the labeling of the subset, which confirms that initial labeling did not have many gross errors.

**Algorithm 6** Human subject consensus.Individual answers by human subjects *L* are transformed to the population answers $L^{*}$. First, calculate number of successes and trials. Success is a human label $l_{h}^{j}$ that is equivalent to original label $l_{j}^{\prime }$. A binomial test (for details see [35]) is used to determine consensus label $l_{j}^{*}$ as a population answer. If more than $p_{e}$ human subject consensus exists, consensus label $l_{j}^{*}$ becomes the most frequent label ${\hat{l}}_{j}$, otherwise it becomes the original label $l_{j}^{\prime }$. = denotes definition, ≡ denotes equivalence, and ← denotes calculation.
**Require:**
$L={\left\{l_{h}^{j}\right\}}_{h\leq |P|}^{j\leq |D^{\prime }|},L^{\prime }={\left\{l_{j}^{\prime }\right\}}_{j\leq |D^{\prime }|},p_{e},\alpha$

**Ensure:**
$L^{*}={\left\{l_{j}^{*}\right\}}_{j\leq |D^{\prime }|}$
 1: **for**
$j\in D^{\prime }$
**do** 2:   successes $\leftarrow \sum_{h}^{\left|P\right|}l_{h}^{j}\equiv l_{j}^{\prime }$ 3:   trials ←|P| 4:   ${\hat{l}}_{j}\leftarrow$the most frequent label(${\left\{l_{h}^{j}\right\}}_{h\leq |P|}$) 5:   *p*-value ←binomial test(successes, trials, $p_{e}$) 6:   **if**
*p*-value < α
**then** 7:    $l_{j}^{*}\leftarrow {\hat{l}}_{j}$ 8:   **else** 9:    $l_{j}^{*}\leftarrow l_{j}^{\prime }$ 10:   **end if** 11: **end for**

**Figure 5:**
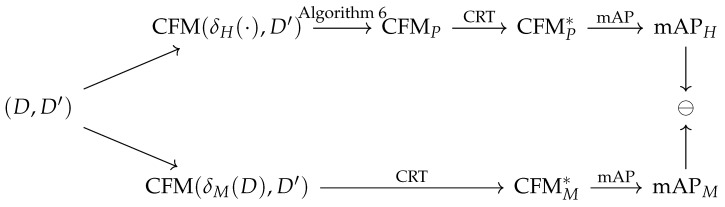
The data processing diagram of user population re-targeting. Dataset is denoted by *D* its subset is denoted by $D^{\prime }$, confusion matrix computed from human results is denoted by $\mathrm{CFM}(\delta_{H}\left(\;\cdot \;\right),D^{\prime })$ and confusion matrix computed from machine classification is denoted by $\mathrm{CFM}(\delta_{M}\left(D\right),D^{\prime })$. ${\mathrm{CFM}}_{P}$ represents population confusion matrix, ${\mathrm{CFM}}_{P}^{*}$ denotes population confusion matrix transformed by CRT operations, ${\mathrm{CFM}}_{M}^{*}$ is a transformed machine confusion matrix, mAP denotes mean average precision metric, and the comparison of metrics is denoted by ⊖.

Given the population and machine classification results on a dataset *D*, it can formally be defined how the CRT influences confusion matrices (see diagram in Figure 5). An untrained classification map is denoted by δ:H→C where H is a set of images and C is a set of classification classes. The testing sets are denoted by D⊂H×C, and a trained classifier on such testing set by δ(D). As human classification is not trained from any test set involved in these experiments, a trained human classifier is denoted by $\delta_{H}\left(\;\cdot \;\right)$. In particular, the machine classifier is denoted by $\delta_{M}$, and human classification is denoted by $\delta_{H}$. Since a confusion matrix is computed from a trained classifier δ(D) on a testing subset $D^{\prime }$, it is denoted by $\mathrm{CFM}(\delta \left(D\right),D^{\prime })$. The comparison of results of mAP (mean average precision) among human and machine classification is denoted by ⊖.

## 5. Experiments

Two similar experiments on different datasets were conducted. First, the ILSVRC2012 dataset (http://image-net.org) was used since it was widely used in the past seven years, and as far as it is evident, never put to scrutiny the way it was done in this work. The population *P* in this experiment was very diverse, subjects from six English-speaking countries with no additional control. Additionally, this experiment was used to verify whether the obtained CRT is only a statistical fluke, and whether people actually like new categories.

A human studies methodology was also used in the VireoFood-172 dataset (http://vireo.cs.cityu.edu.hk/VireoFood172). The dataset is not so widely used and it is also much smaller in size. Because it contains only Asian food, it is somewhat ideal for experimenting on a more *specific* population that would result in stronger effects. For this experiment, two different populations ENG and ASIA were used. The ENG population consisted of subjects from two English-speaking countries, and the ASIA population contained subjects from two Asian countries.

### 5.1. Materials

Reduction in the dataset size (Algorithm 2) and determining population size (Algorithm 3) were performed with RStudio version 1.1.442 [36], R version 3.3.2 [37] and pwr package version 1.2-0 [38]. Additionally, the results were cross-checked with G* Power 3 version 3.1.9.2 [39].

For human studies (see Section 3.2), subjects were recruited using the Clickworker (http://www.clickworker.com) platform. The experimental environment was arranged on a custom server, where each subject received a unique link to a sequence of tasks, which they solved in the web browser. For the experimental environment, Django version 2.1.7 with Python version 3.6.7 was used and it was running on Nginx server version 1.16.0. Data was saved in the Postgresql database version 11.3.

For the clustering method in Algorithm 4, the Python package kmodes version 0.10.1 [40] was used as the implementation of the k-Modes algorithm [41,42] with the improved initialization method [43]. A number of clusters *k* was defined by the elbow method. The algorithm was run with different numbers of *k* to get the graph of the cost function P(W,Q) where *W* is a partition matrix and Q is a vector of modes (for details see [41,42]). The optimal *k* was selected on the location of an elbow (see Section 6.1 and Section 7.3 for details).

When determining renaming operations by Algorithm 5, the SymSpell algorithm [44] was selected for word segmentation. For the implementation of the algorithm, a Python package symspellpy version 6.3.8 [45] was used. Newly created words were then tokenized by NLTK version 3.4.3 [46]. The lemmatization process was also done by NLTK using WordNet [47]. Non-existent lemmas were replaced by ‘unknown’. For other cleaning functions, standard Python version 3.6.7 procedures were used.

MXNet version 1.5.0 and GluonCV framework version 0.5.0 [48,49] were used for deep learning models. Evaluation and comparison of population and machine classification was done using Python packages Pandas version 0.24.2 [50], Scipy version 1.3.1 [51], Scikit-learn version 0.21.2 [52], and Numpy version 1.17.0 [53,54].

### 5.2. Experiments on ILSVRC2012 Dataset

The ILSVRC2012 object detection dataset [19] was used as the dataset *D*. It contains |C|=1000 categories. Each category contains from 732 to 1300 images. Unfortunately, the ground truth for the ILSVRC2012 test images was not available. For all tests, ILSVRC2012 validation images were used. While this did not influence human performance, it is plausible that it overestimated machine performance to a certain extent.

#### 5.2.1. Reduction of Dataset Size

The preliminary testing suggested that on average, the maximum time for a subject to still have enough concentration to end the task is 20 min. Based on the experience with preliminary test subjects (did not participate in the main experiment), it was established that it would be appropriate to have $\nu_{c}=5$ categories and $\nu_{i}=8$ images per category. This totals in 40 images per batch *B*. See Section 3.1 for definitions.

To detect large changes between the smaller experimental dataset $D^{\prime }$ and the original dataset *D* at least $|C^{\prime }|=10$ categories, $|H^{\prime }|=16$ images per category, and |B|=4 batches were needed. The numbers were obtained by Algorithm 2 from Section 3.1 with parameters |C|=1000, $\nu_{c}=5$, $\nu_{i}=8$, $h_{c}=0.9$, $h_{i}=0.5$, α=0.05, and 1−β=0.8.

#### 5.2.2. User Studies

For proper user studies, at least |P|=92 subjects and $n_{o}=23$ observations per image were required. The numbers were obtained by Algorithm 3 from Section 3.2 with parameters |B|=4 batches, $p_{m}=0.5$, $p_{e}=0.75$ and 1−β=0.8.

Please note, $n_{o}=23$ observations per image is a number that is needed for statistical significance. It is much larger than the number of observations usually used when annotating computer vision datasets!

The only constraint placed on the population of subjects *P* in user studies was that they live in the countries where the primary language is English (the UK, the USA, Canada, Australia, Ireland, New Zealand), as Task 2 requires text entry of labels, which assumes familiarity with the English language. Each subject was paid 3 EUR for 20 min of work to motivate them to approach the tasks seriously. In practical applications, the human subjects would be sampled according to some predefined criteria (e.g., sampled from a target market or a target demography for an AI-powered machine).

#### 5.2.3. Choice Of Categories

*Eight* ILSVRC2012 categories that have the lowest frequency (popularity) in English texts were chosen to simulate a *challenging* task for human observers. As a control, *two* categories that are most frequent in a written text were added. When choosing eight low-frequency categories, those that have a high-level concept in their names were skipped. It is obvious to humans that <frilled lizard> is a kind of a lizard, and in the absence of multiple lizard species, such results would not reflect the ability of humans to correctly classify categories they never heard of. The categories are shown in Table 1.

#### 5.2.4. Deep Learning Models

For comparison of humans to the machine, widely known AlexNet [55], VGG19 [56], and ResNet152v2 [57] were used. All of them were pretrained on the ILSVRC2012 dataset. AlexNet is an older architecture and it was used on purpose, to give it a better chance in the improvement of performance between original dataset categories, and the new, CRT-transformed categorization.

### 5.3. Experiments on VireoFood-172 Dataset

**Figure 6:**
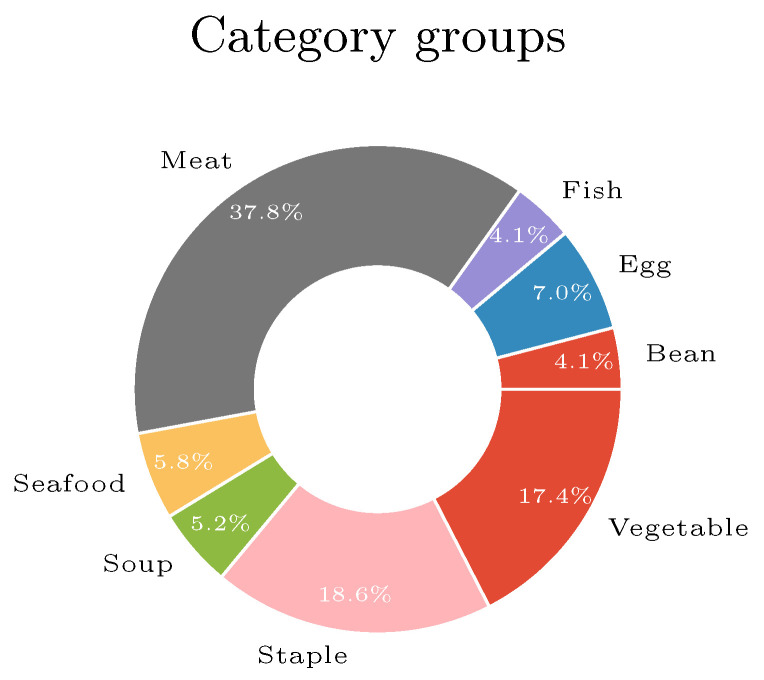
Distribution of food categories under eight groups from VireoFood-172 dataset [58].

The VireoFood-172 dataset [58] was also used as the dataset *D*. It contains |C|=172 categories of popular Asian dishes. Each category contains from 191 to 1061 images. The categories cover eight groups of foods Bean products, Egg, Fish, Meat, Seafood, Soup, Staple, and Vegetables. The distribution of food categories under eight groups is represented in Figure 6.

#### 5.3.1. Reduction of the Dataset Size

Following the methodology from Section 3.1, at least $|C^{\prime }|=16$ categories, $|H^{\prime }|=18$ images per category, and |B|=6 batches were needed to detect large changes between the smaller experimental dataset $D^{\prime }$ and the original dataset *D*. The numbers were obtained by the Algorithm 2 with parameters |C|=172, $\nu_{c}=8$, $\nu_{i}=6$, $h_{c}=0.8$, $h_{i}=0.5$, α=0.05, and 1−β=0.8.

#### 5.3.2. User Studies

For user studies (see Section 3.2), $n_{o}=24$ observations per image and |P|=144 subjects were required. The numbers were obtained by Algorithm 3 with parameters |B|=6, $p_{m}=0.5$, $p_{e}=0.75$, and 1−β=0.8.

In the case of the VireoFood-172 dataset, two experiments with different populations of subjects were performed. The first population of 144 subjects $P_{1}$ lives in the UK or Ireland. With such a constraint, a large pool of culturally similar subjects were obtained.

The second population of 144 subjects $P_{2}$ only live in Malaysia or Singapore. The constraint also brought a large pool of culturally similar subjects. Note that using the Clickworker platform, there were limited options for Asian countries to select. China was not among them.

The age constraint (18–50) was set to both populations. With the age constraint, experiments were focused on the population, which was expected to be more computer literate.

Each subject was initially paid 2.25 EUR for 15 min of work, to motivate them to approach the tasks seriously. To be able to recruit enough subjects from Ireland and Singapore, the payment was gradually increased to 8 EUR.

#### 5.3.3. Choice of Categories

Before choosing the categories, a demography experiment was conducted where the subjects from the chosen population were asked about their eating habits. Both populations were mainly omnivores (they eat everything). By also considering the distribution of category groups (Figure 6), the most representative categories would be the ones from the meat group. From this group, 16 random categories were chosen, which are shown in Table 2.

#### 5.3.4. Deep Learning Models

For comparison of humans to the machine, the same pre-trained models from the ILSVRC2012 experiment were used. The models were fine-tuned on the VireoFood-172 dataset. The models were fine-tuned with the parameters momentum 0.9, weight decay 0.0001, base learning rate 0.001 with decay each 10th epoch by the factor 0.75, and 60 training epochs were used.

The data for training was augmented in the following way. First, the original image was cropped with a random size 0.08 to 1.0 and a random aspect ratio 0.75 to 1.33, and then resized to 224. Next, the image was randomly flipped left or right with a probability of 0.5. Image brightness, contrast, saturation, and hue were randomly jittered by a factor 0.4. Then, AlexNet-style PCA-based noise was added to the image with an intensity of 0.1. Finally, the image was normalized with ImageNet mean (0.485,0.456,0.406) and standard deviation (0.299,0.224,0.225).

## 6. ILSVRC2012 Results and Discussion

**Figure A1:**
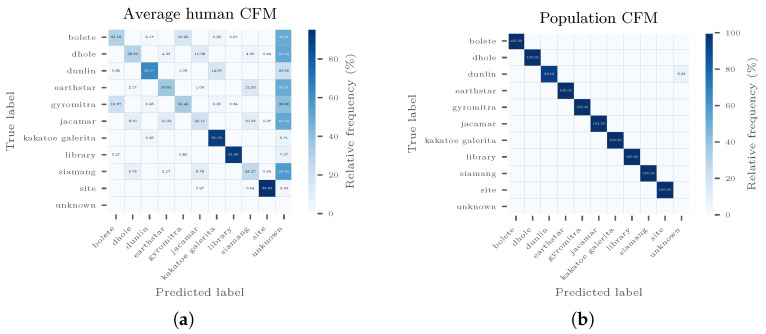
Obtained confusion matrices (CFM) for human subjects from the ILSVRC2012 experiment. The matrix in (**a**) represents averaged results for all human subjects. (**b**) shows a population CFM.

CFM analysis has shown human subjects created many false positive and false negative results for each category (see Figure A1 in the Appendix A for details). Also, many images, regardless of the category, were classified as <unknown>. This suggests the classification task was hard as subjects did not know which category to choose. <site> category had the best result as it was correctly recognized 96% of the time. Also, <kakatoe galerita> and <library> were easily recognizable. That the <site> and <library> were one of the most recognizable categories, is consistent with the hypothesis that the popular categories will be easily recognized by humans (see Section 5.2.3). In contrast, the ability to separate other classes, excluding <kakatoe galerita>, was very low in humans. Metrics Top-1 ACC (top-1 accuracy), Precision, Recall, and F1-Score were calculated 51.4%, 65.2%, 46.8%, and 53.0% respectively. The numbers are very low, but that does not necessarily mean people are bad at classifying. The results rather indicate that given labels cannot be correctly associated with given images by more than 75% of the population.

There is a significant difference between the average human confusion matrix ${\mathrm{CFM}}_{H}$ (Figure A1a), obtained by averaging categorization results of $n_{o}$ unique human subjects, and the population confusion matrix ${\mathrm{CFM}}_{P}$ (Figure A1b), which was determined by the methodology from Section 4. ${\mathrm{CFM}}_{H}$ results show there were many outliers, and with human subject consensus (Algorithm 6) they were successfully eliminated. For example, the algorithm removed the potential answers from humans that were not taking the experiment seriously (randomly selecting the answers) or answers that were selected by mistake. These kinds of errors cannot be successfully supervised with online experiments.

**Figure A2:**
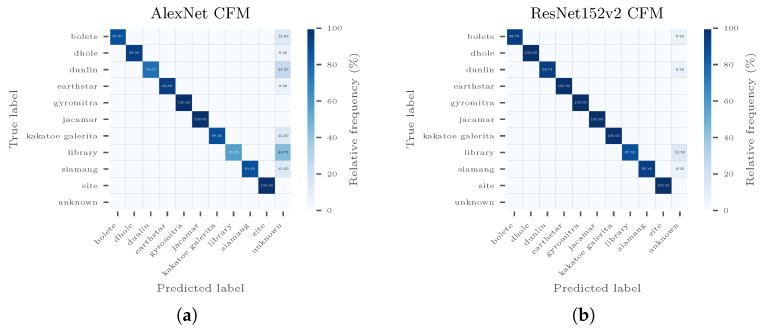
Obtained confusion matrices for CNNs from ILSVRC2012 experiment. The matrix in (**a**) was derived from AlexNet classification results. (**b**) is ResNet’s CFM.

The machine confusion matrices ${\mathrm{CFM}}_{M}$ were obtained simply by running respective algorithms on the validation set of the selected ILSVRC2012 categories. AlexNet, the oldest CNN architecture had the worst classification results (Figure A2a). The best CNN results were obtained by ResNet architecture (Figure A2b). CNN metric results can be viewed in Section 6.2. Note that the term ‘original’ is strictly used for CFM matrices before applying CRT and ‘changed’ for transformed CFM matrices that have gone through a CRT transformation.

The comparison of ${\mathrm{CFM}}_{P}$ and ${\mathrm{CFM}}_{M}$ implies CNNs are not superior in the classification task. Humans outperformed all CNN models as later classified more images into the <unknown> category.

It should be noted that human and machine error modes are somewhat different: humans will label the image as <unknown> if they do not know the answer, but the machine, pre-trained on ILSVRC2012 will choose the label outside of selected categories from $D^{\prime }$. Both of these categories were denoted as <unknown> even though they represent conceptually different things. This is necessary to keep the human experiment within the manageable limits.

### 6.1. Deriving CRT operations

**Figure 7:**
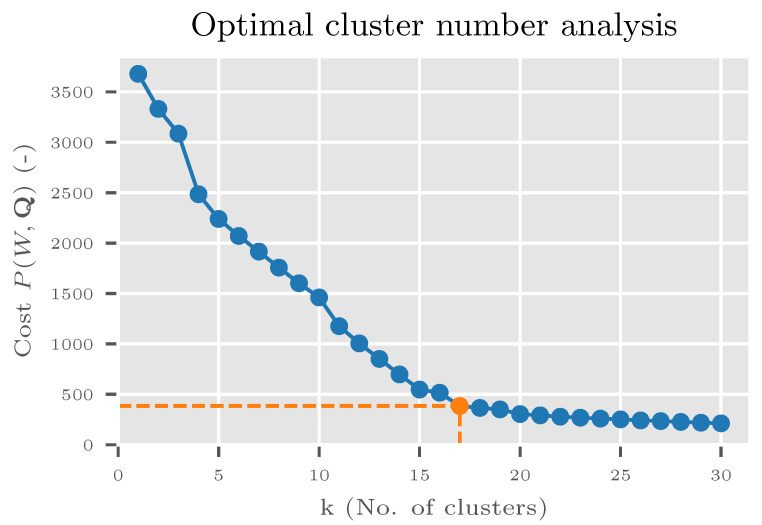
Optimal cluster number analysis for ILSVRC2012 experiment. An analysis was done by the k-Modes algorithm [41,42,43]. A clear change of slope is visible at 17 clusters (orange markings).

A total of 17 optimal clusters were determined by the elbow method and cost function P(W,Q) (Figure 7). There was a clear change of slope at 17 clusters and a noticeable drop after three clusters. It was verified that the three clusters did not provide any meaningful clustering, and the value of 17 was used.

The results indicate that even though humans created a larger amount of clusters, the most frequent labels were sometimes the same for multiple clusters. This was an important revelation: people *knew* that there were two different kinds of birds in images, but they were OK if they got the same label. These labels were merged to get more general categories.

Transformation of the dataset $D^{\prime }$ into a new one is represented by Table 3. All bird types were merged into <bird> category and all fungi types were merged into <mushroom> category. This was consistent with the results from Task 3 where the population CFM was obtained. The matrix indicated bird and mushroom category types as one of the hardest categories to distinguish.

<siamang>, <dhole>, and <site> were renamed to <monkey>, <fox>, and <website> respectively. The results indicate that the users did not associate the dataset-provided labels with the corresponding images. Thus, more general labels should be considered. Even more, users thought that images of <dhole> category depict a fox. In reality, <dhole> is a wild dog and despite being in the same taxonomic family, Canidae does not fit into the subfamily of a fox. Nevertheless, <dhole> *visually* looks like a fox, and that is the most obvious explanation for the observed change in labeling.

<library> was split by users into the categories <book> and <library>. Upon examination of the data, it was found that the original category was quite heterogeneous—some images were more easily associated with the <book> rather than the <library>. Labels are conceptually close, as libraries contain books, but there is sufficient ambiguity that it gave rise to the two distinct categories.

Hard to recognize images were put into the new <unknown> category. This category represented the images that were hard to recognize or hard to name.

### 6.2. Human Population and Machine Classification after Applying the CRT

**Figure A3:**
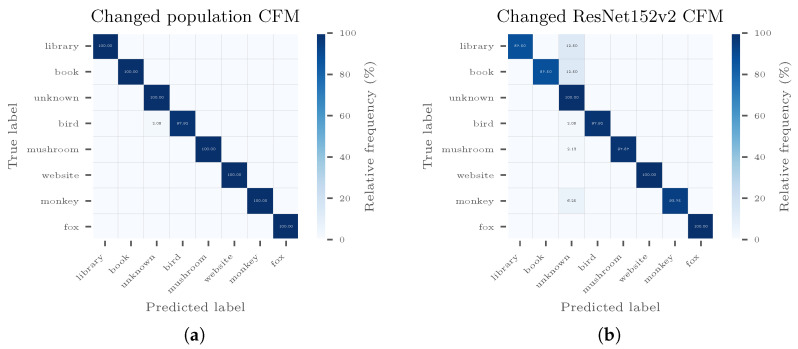
The human population CFM (**a**) and ResNet CFM (**b**) after applying CRT to the dataset $D^{\prime }$, which was derived from the ILSVRC2012 dataset.

The Top-1 ACC metric of population results did not change and neither did they for the ResNet model. A significant change was perceived in all other metrics. The precision of human results increased by 6.0 pp (percentage points), but the precision of ResNet dropped by 1.3 pp (Table 4). The increase of human results clearly shows the importance of choosing correct category labels and correct images that are associated with those labels. Similarly, the drop in the precision metric for CNN indicates that their performance can be overestimated if a dataset is not constructed with a human population in mind. Note, transformed CFM matrices ${\mathrm{CFM}}_{P}^{*}$ and ${\mathrm{CFM}}_{M}^{*}$ can be viewed in Figure A3a and Figure A3b respectively.

In the recall metric human results increased by 9.4 pp and ResNet’s increased 7.5 pp. Similarly, there was an 8.2 pp increase from original to changed in human’s F1-Score, and 0.7 pp drop in ResNet’s F1-Score.

Similar results of the CRT transformation were obtained for other computational models too. The results can be seen in Table 4 for VGG19 and AlexNet as well. They are consistent with the results on ResNet as Precision dropped, Recall increased, F1-Score dropped as well, and Top-1 ACC did not change.

### 6.3. Qualitative Results

**Figure 8:**
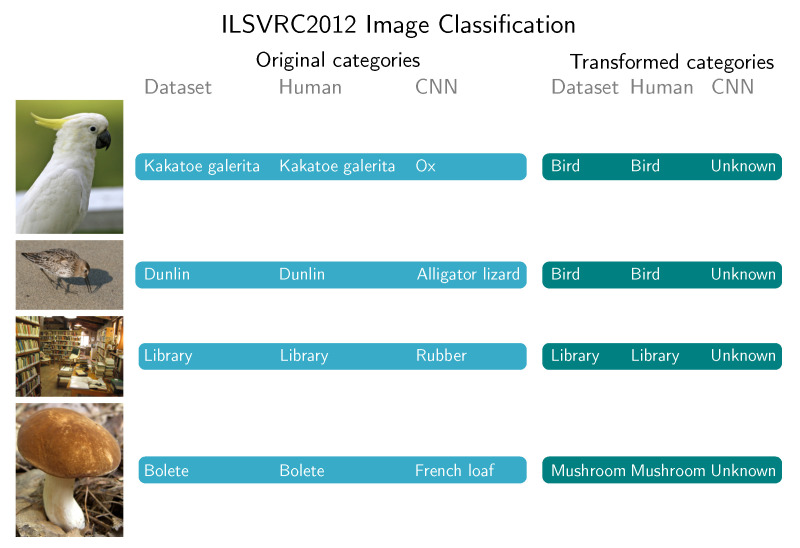
Classification on ILSVRC2012 dataset [19]. When using original categories CNN classifications are absurdly different from true categories. Images are only examples to visually represent a dataset category. They are not part of the dataset. <kakatoe galerita> photo by Lip Kee Yap licensed under CC BY-SA 2.0, <library> photo by Christopher John SSF and <bolete> photo by Jason Hollinger licensed under CC BY 2.0.

Four examples of images where CNN clearly missed the category are shown in Figure 8. The first image represents <kakatoe galerita> and was correctly recognized by humans. AlexNet recognized it as <ox>. This is not the kind of mistake that humans would make, and consequently, the CNN can be hardly defined as intelligent in this case. Such mistakes could potentially evoke frustration in users of the real appliance, relying on the same CNN method.

### 6.4. Verification

**Figure 9:**
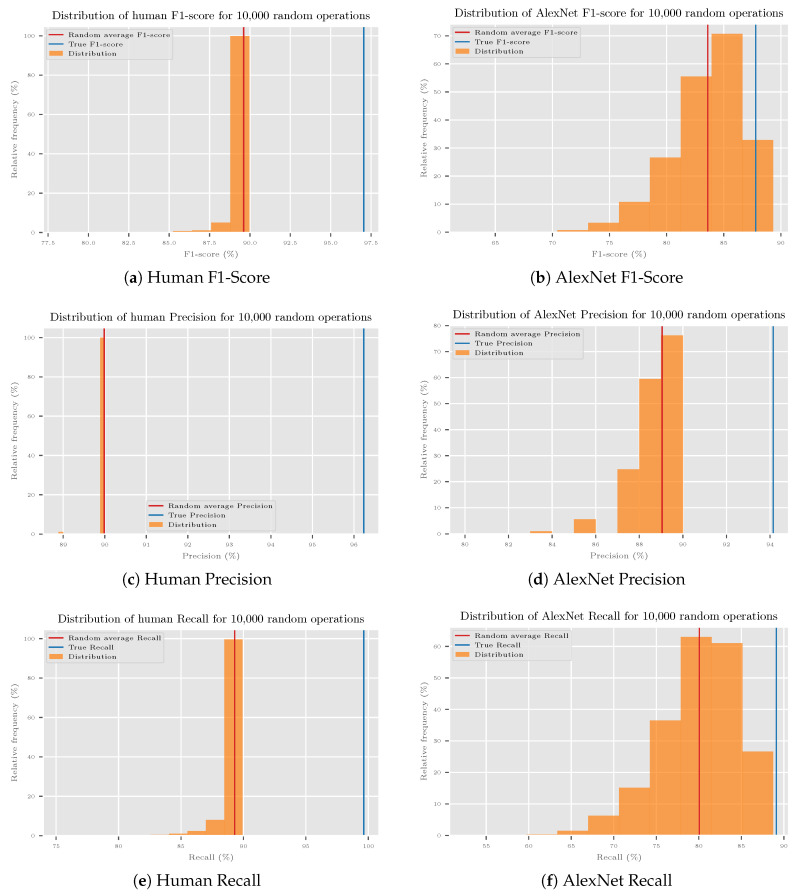
Changed human (**a**,**c**,**e**) and machine (**b**,**d**,**f**) metrics (in blue) on ILSVRC2012 subset are not accidental, since almost all randomly generated CRTs performed worse in the case of human and machine (orange bars represent results of random test in histogram form, heights are bin frequencies). Note that almost all orange bars are to the left of the blue line.

The first question is, whether the changed metrics are only a statistical fluke. To examine this, 10.00 random CRT transforms were generated, which all contained nine final categories, and observed the range of possible metrics for human and machine. The results, which are shown in Figure 9, were evaluated by the Wilcoxon signed-rank test as distributions. The null hypothesis was defined as *Performance of random CRT transforms is equal to measured performance for humans and a machine*. The null hypothesis was rejected as *p*-values for human and CNN results were >0.001. The obtained CRT is, thus, very likely not a statistical fluke.

**Figure 10:**
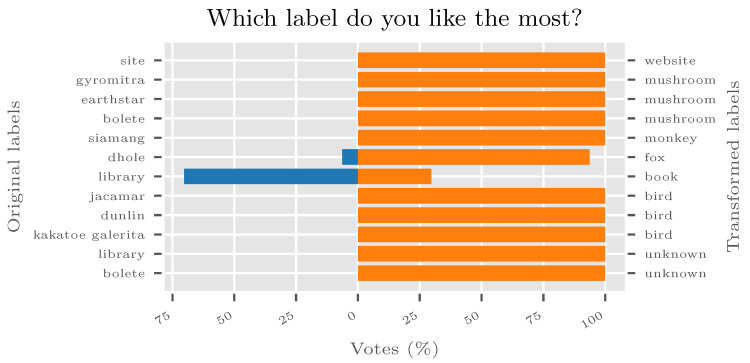
Results of the final verification of the ILSVRC2012 experiment. Even if the disjointed sample of the population is tested, they still prefer CRT-transformed categorization by a large margin. The blue bars denote the percentage of votes for the original category, and orange bars denote votes for the transformed category. Note that most of the previous categories did not get even a single vote.

The final question is, whether people actually *like* new categories. The new human verification test was set up with subjects who did not participate in any of the previous tests but have been drawn from the same population. They were presented with images, and the choice of pre-CRT label and post-CRT label for the image. One hundred people were asked simply “Which label do you like the most?” and all images from $D^{\prime }$ have been reevaluated this way. The results, shown in Figure 10, are conclusive—*people prefer CRT-transformed categories by a large margin*.

## 7. VireoFood-172 Results and Discussion

Different from the previous experiment, this experiment involved two distinct populations of human subjects. The first population of 144 subjects was recruited from the UK and Ireland and denoted ENG. Their mean age was 31.45 ± 8.54. Of the total, 48% of participants were female and 52% were male; 67% of participants had normal vision, 24% had mild vision loss, and 9% moderate vision loss. The second population of 144 subjects was recruited from Malaysia and Singapore and denoted ASIA. Their mean age was 29.34 ± 7.69. Of the total, 45% of participants were female and 55% were male; 62% had normal vision, 27% had mild vision loss, 11% had moderate loss; and 0.6% had severe loss of vision.

**Figure 11:**
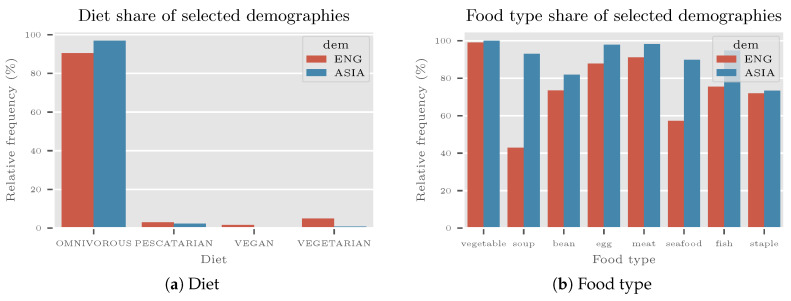
Diet (**a**) and food type (**b**) demographic data of the VireoFood-172 experiment. More than 80% of all subjects were identified as omnivorous and ate meat at least once in the last week.

Their self-identification by their diet is shown in Figure 11a. ANOVA statistical power analysis for diet groups (effect size h=0.4, α=0.05, statistical power 1−β=0.8, and 4 groups) has shown that at least 76 subjects were needed to correctly identify the diet of the population. As 144 subjects for each demography were used, the statistical power for the ANOVA test rose to 0.99.

More than 80% of all subjects were identified as omnivorous. To test the significance of the diet preference a $\chi^{2}$ test of independence with null hypothesis *diet preference is not associated with demography* was used. The hypothesis was rejected for both categories as the *p*-value was >0.001. The result shows that the population was appropriate to use in the experiment, as a significant amount of people eat meat.

Figure 11b represents answers about what did people eat in the last week at least once. The biggest differences between ASIA and ENG demography have been observed for soup, seafood, and fish food types, which is not considered a problem, as these categories were not considered in the experiment. Regarding meat, more than 80% were eating it in the last week. This also indicates suitability for using the selected populations in the experiment.

### 7.1. Training CNNs

**Figure 12:**
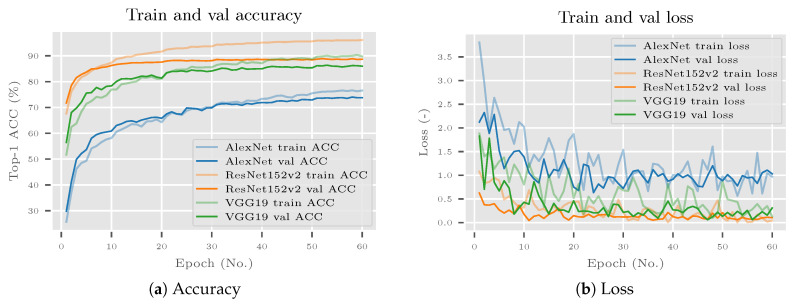
Accuracy (**a**) and loss (**b**) graphs of training and validating CNNs on VireoFood-172 subset. All graphs show relatively well-trained CNNs. The algorithms did not overfit, and additional training would not significantly improve the performance.

Differently from the previous experiment, CNNs were additionally fine-tuned on the VireoFood-172 dataset. Training and validation accuracy for selected CNNs is shown in Figure 12a. Validation accuracy rose above train accuracy for all algorithms and had settled down by 60 epochs. As validation accuracy did not start to fall, the algorithms did not overfit. An additional confirmation for that are loss charts (Figure 12b). Validation loss was nearly the same as train loss, but it did not start to rise. Based on this analysis it can be concluded that the deep neural networks were trained relatively well. As accuracy did not change much from epoch 50 onwards, it was assumed that additional training would not improve the performance by a significant margin.

### 7.2. Pre-CRT Results

**Figure A4:**
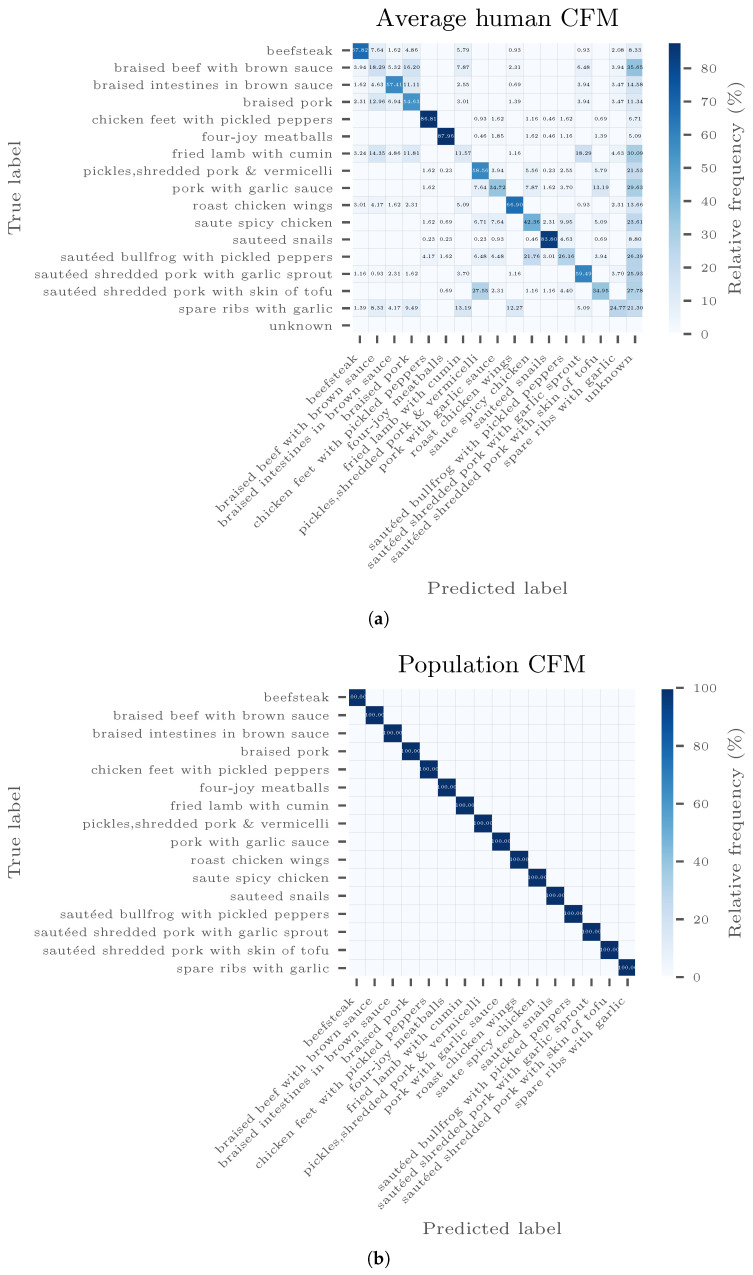
Average human (**a**) and population (**b**) CFMs for the ASIA population of the VireoFood-172 subset.

**Figure A5:**
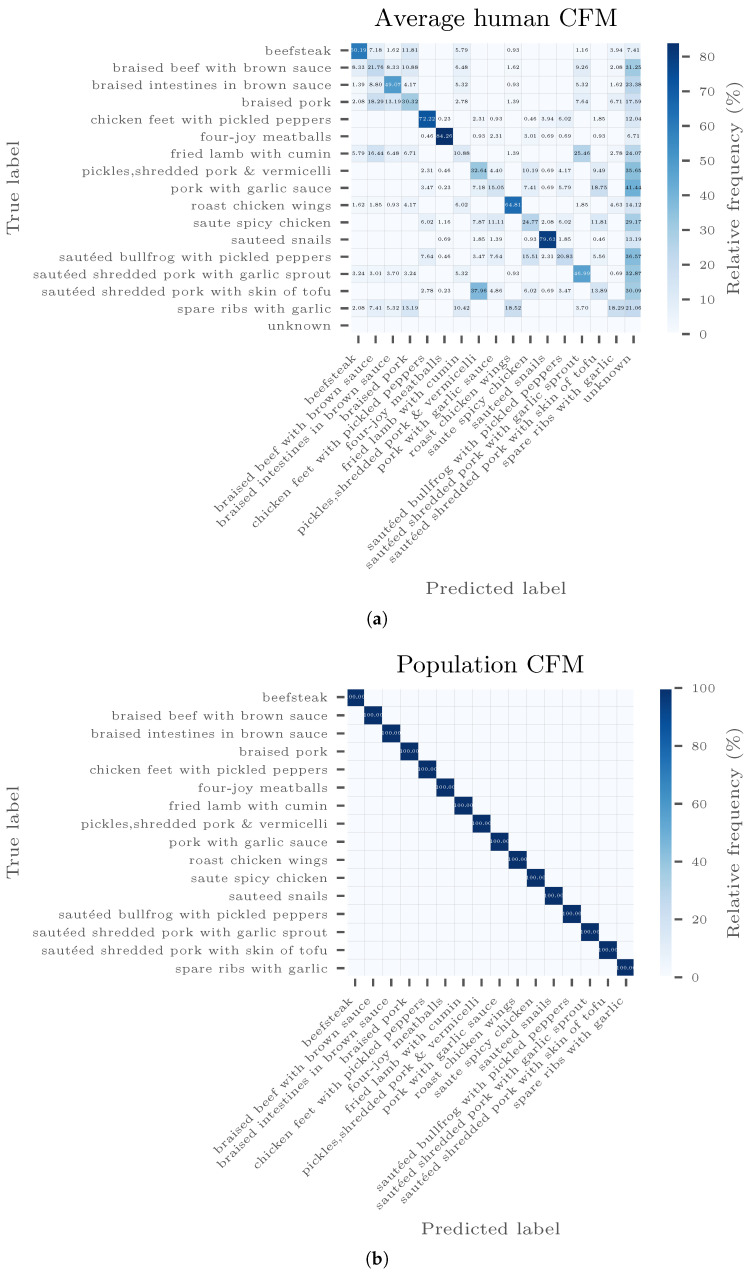
Average human (**a**) and population (**b**) CFMs for the ENG population of the VireoFood-172 subset.

Original CFMs for ASIA population (Figure A4a in Appendix B) and ENG population (Figure A5a), obtained by subject categorization, have shown quite dispersed classifications with many mistakes. People disagreed to a certain extent as to which label should be appended to which image. Top-1 ACC, Precision, Recall, and F1-Score for ASIA population were 51.0%, 57.4%, 48.0%, and 51.7% respectively. This indicates a degree of disagreement among the human population. The results were slightly worse than the ones from the ILSVRC2012 experiment. This shows that the classification task was harder. Images are visually very similar and the type of meat is difficult to recognize from the image. Top-1 ACC, Precision, Recall, and F1-Score for ENG population were 40.4%, 47.0%, 38.0%, and 41.4% respectively. The original results for the ENG population were even worse. This was expected as food is only Asian and is considered harder to recognize by non-Asian subjects.

Using the human subject consensus (Algorithm 6 from Section 4), both CFMs (Figure A4b and Figure A5b for ASIA and ENG population respectively) became diagonal matrices after adjusting them for population consensus. The analysis concluded there is no category, where more than 75% of the population would agree on a label change. This is true for both populations.

**Figure A6:**
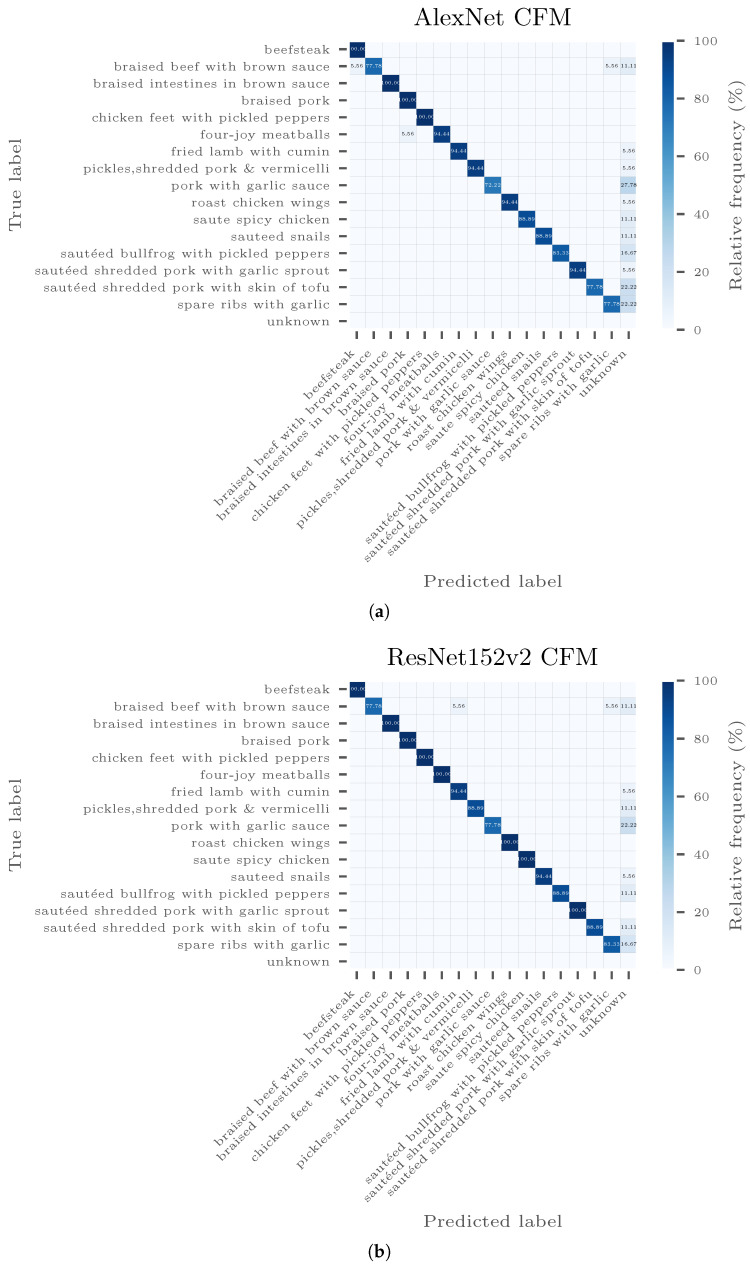
AlexNet (**a**) and ResNet (**b**) confusion matrices in VireoFood-172 experiment.

CFMs for AlexNet and ResNet deep neural networks (Figure A6) were very similar to each other, but metrics (see Section 7.4 for details) show ResNet outperformed AlexNet. Compared to ASIA and ENG human results, neural network performances on the original subset were worse. In addition to mistakes, many images were classified into categories that were out of the scope of the experiment and thus fell into the <unknown> category. The worst results for ResNet were obtained for <pork with garlic sauce>, where 22% of images were classified as <unknown>.

### 7.3. Deriving CRT Operations

**Figure 13:**
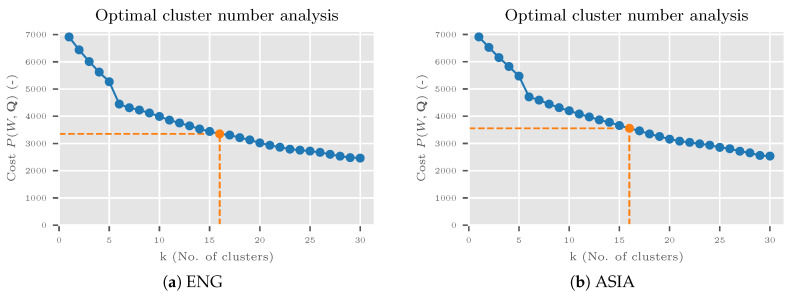
Optimal cluster number analysis for the VireoFood-172 experiment. The analysis was done by the k-Modes algorithm for ENG population (**a**) and ASIA population (**b**). Sixteen clusters were selected (orange markings).

Sixteen optimal clusters were determined by the elbow method and by cost function P(W,Q) for both ASIA and ENG demography (Figure 13). The change in slope for selected points was not very noticeable as it was for six clusters, but the cost was significantly lower. The selected values also corresponded to the original number of categories.

As before, despite creating 16 clusters, human labels were sometimes the same, so the clusters were merged and got 10 different categories for ENG population, and seven different categories for the ASIA population. The finding was surprising as more categories were suspected coming out of the ASIA population. Because the food represented was Asian, it was assumed ASIA subjects would have an easier time separating different dishes.

The differences of categories for each demography can be shown in Table 5 and Table 6. Both populations defined <meat>, <vegetable>, <meatball>, and <noodle> as new categories. They contained similar images. ASIA population additionally defined <intestine>, <pork>, and <seafood> categories that were not present in ENG population. In contrast, <chicken foot>, <fry stir>, <soup>, <pasta>, <snail>, and <stew> were defined by ENG population and were not present in ASIA population.

### 7.4. Post-CRT Results

**Figure A7:**
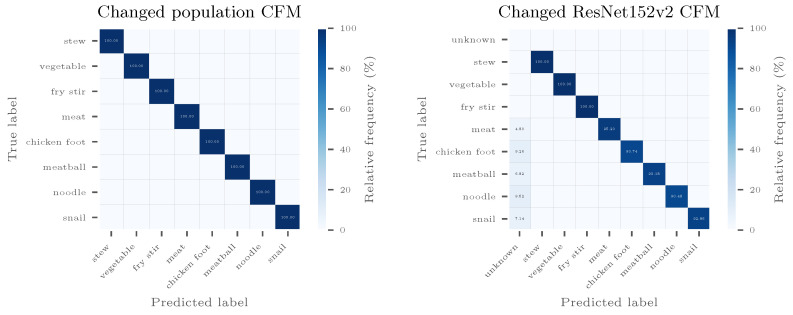
The population (**a**) and ResNet (**b**) CFM from after applying CRT. CFMs are obtained from the ENG population and VireoFood-172 subset.

After transforming ${\mathrm{CFM}}_{P_{1}}$ (ENG demography) and ResNet ${\mathrm{CFM}}_{M}$ (see Section 3 for details), the ${\mathrm{CFM}}_{P_{1}}^{*}$ (Figure A7a) remained the perfect diagonal matrix. ResNet ${\mathrm{CFM}}_{M}^{*}$ (Figure A7b) also stayed similar at a glance, but the metrics in Table 7 shows there was a change in performance. Top-1 ACC increased by 0.7 pp, Precision dropped by 4.5 pp, Recall also dropped by 3.2 pp, and F1-Score dropped by 3.7 pp. Similar results were obtained from other CNN models. It was also observed that ${\mathrm{CFM}}_{P_{1}}^{*}$ does not have any <unknown> category, but ResNet’s does. This shows that humans identified all images by new categories and ResNet categorized some of the images to the categories that are out of the scope of the dataset $D^{\prime }$.

**Figure A8:**
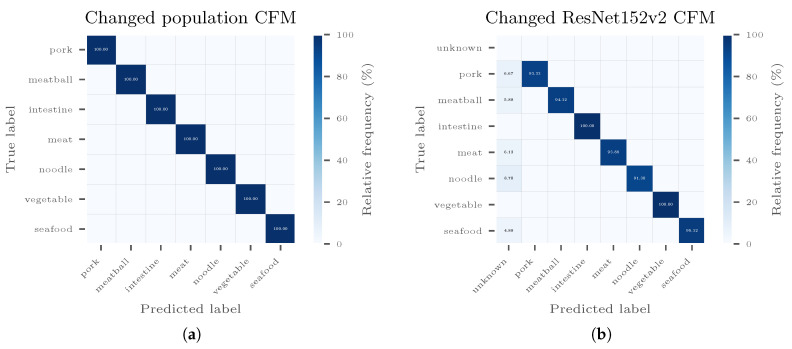
The population (**a**) and ResNet (**b**) CFM after applying CRT. CFMs are obtained from the ASIA population and VireoFood-172 subset.

Similar to transformations for the ENG population, ${\mathrm{CFM}}_{P_{2}}^{*}$ (Figure A8a) from the ASIA population was diagonal and did not contain an <unknown> category. ResNet ${\mathrm{CFM}}_{M}^{*}$ (Figure A8b) stayed similar at a glance. ResNet’s Top-1 ACC (Table 8) was the same, but Precision, Recall, and F1-Score were lower than with the ENG population by 1.4 pp, 1.3 pp, and 0.8 pp, respectively.

### 7.5. Qualitative Results

**Figure 14:**
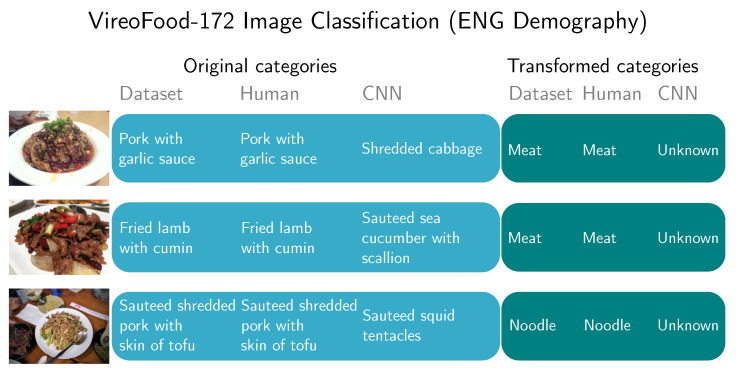
Classification of selected images from VireoFood-172 dataset [58] by ENG population. When using original categories CNN classification are different from true categories. Images are only examples to visually represent a dataset category. They are not part of the dataset. <pork with garlic sauce> photo by Irrational cat and <fried lamb with cumin> photo by Alchen_x licensed under CC BY-SA 2.0, <sauteed shredded pork with skin of tofu> photo by T. Tseng licensed under CC BY 2.0.

**Figure 15:**
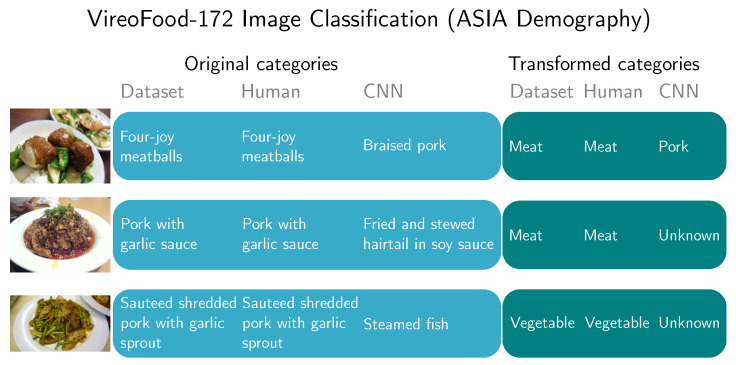
Classification of selected images from VireoFood-172 dataset [58] by ASIA population. When using original categories CNN classifications are different from true categories. Images are only examples to visually represent a dataset category. They are not part of the dataset. <four-joy meatballs> photo by Yoppy licensed under CC BY 2.0, <pork with garlic sauce> photo by Irrational Cat and <sauteed shredded pork with garlic sprout> photo by Kent Wang licensed under CC BY-SA 2.0.

Examples of images where CNN clearly missed the category are shown in Figure 14 and Figure 15. The first image in Figure 14 represents classification of a <pork with garlic sauce> in ENG demography. Humans correctly classified it, but AlexNet CNN classified it as <shredded cabbage>, which is clearly incorrect, as only the meat supercategory was used. By transforming human and CNN predictions into new categories, humans still perfectly categorized the image. CNN now changed its result value to <unknown>. From a user’s perspective, it is considered a better result.

The appearance of ‘exotic’ foods (<chicken foot>, <snail>) after performing the CRT transformation that was derived using the ENG population was a clearly unexpected result, which was not observed in the ASIA group. According to results on ImageNet, a general trend towards less specific labels was expected, especially in the ENG population, which should be less familiar with Asian food. However, there may be a simple explanation: the ENG population is *sensitive* to the food that contains unfamiliar animals or unfamiliar parts of the animals since they want to *avoid* it—they *want to know* whether the food contains unfamiliar ingredients that may be unpalatable to them. This unpredictable result further emphasizes the importance of user tests, when evaluating classification algorithms.

The first image in Figure 15 represents a classification of a <four-joy meatballs> in the ENG demography. Humans still correctly classified it, but AlexNet CNN classified it as <braised pork>. By transforming human and CNN predictions into new categories, humans still perfectly categorized the image. CNN now changed its result value to <pork>. The reason, why CNNs transformed the label into <pork> is that the original prediction is part of the dataset $D^{\prime }$ labels.

## 8. Conclusions

The majority of the computer vision develops methods on the datasets with hard annotations. Hard annotations are the ones that are normally not changed. However, as this work showed, this may lead to sub-optimal results when such algorithms are used in real-world applications. A frequently heard prognosis that CNNs will soon outperform humans is to be interpreted strictly with the understanding that the current resemblance between evaluation methods and human reasoning and performance is not sufficient.

Some argue that the problem can be addressed by simply adding more data, but this route has its limits. It is unsustainable to use millions of images on models that do not generalize well and will eventually overfit. It should be minded that humans need only a couple of images to learn new concepts. Another possibility is to increase the model capacity, but this is unsustainable as well. There are already CNN models with millions of parameters and more than 100 layers. In many cases, the research resorts to active learning, but the validity of this approach is questionable for practical applications [59]. Active learning is prone to runaway feedback loops or even abuse. In practice, AI-powered devices should be ready for consumer use immediately after purchase, or the customers may feel they are not getting good value for money.

A deeper look into the problem of poor dataset annotations would better address the problem. One of the reasons behind poor annotations is the classical view of the categorization, which assumes that categories are based on shared properties. When creating benchmark datasets, the categories are normally chosen according to narrow research goals. Researchers obtain a huge amount of images (possibly from the internet) and label them as they see fit. However, newer research shows that categorization is, in fact, a matter of both human experience and imagination [18]. The preferred categorization between people with different experiences and cultures will be different. Therefore, it is essential to introduce the concept of a *target user* in popular benchmarks and performance tests. Category selection is, therefore, not a trivial task.

How to decide which possibility of categorization fits the target user population? Which one categorization will encourage algorithms to learn the answers that will *not frustrate* the target users? To answer these questions, this work has presented a novel method for the transformation of categorization tasks. The method can be used to transform a categorization problem, defined by the dataset and human as a target-user, into the problem (dataset) that is more *relevant* for the target population. A sufficient number of samples have to be used to make the results statistically significant—this is a topic that is often overlooked in the comparison between human subjects and automated recognition algorithms.

### Summary of Findings

• *The performance of human observers on ImageNet problem is probably better than previously thought.*

In [19] they have reported 94.9% Top-1 ACC, but this research has measured 99.4% Top-1 ACC. Note that there is a significant difference in the methodology. In [19] the metric was estimated from one “trained” human, who annotated 1500 images. A human was trained on 500 sample images. This work, on the other hand, used a much more sophisticated methodology. True classification tasks were used, where a human can choose among a set of categories and not only an annotation task which is similar to the labeling task. Using a classification task provides equal treatment of people and machines and is much more appropriate for comparison of humans to machines. *Training* humans, as done by [19], does not reflect the human experience and reasoning—it assumes the actual users of AI will yield to the methodology of AI community, and not vice-versa. Twenty-three participants per image were used and not only one, to properly sample experimental populations. The result of this work is, therefore, statistically significant and adapted to a selected population. The methodology of [19] did not even consider the question of different populations, and thus, their result could vary significantly if they would use different human annotators. Also note that their annotator labeled 1500 images, but each of the participants in this paper classified only 40 images, which is a considerable difference regarding human fatigue.

• *The performance of CNNs is target user population-dependent.*

The performance of CNNs changed in almost all cases after the *user population re-targeting* (that is, applying the CRT). The changes are especially striking in the VireoFood-172 experiment when CRT was derived from the ASIA population. It should be minded that improvements of just a few percentage points over the state-of-the-art provide good chances of acceptance to most competitive computer vision conferences, but here it is demonstrated that 7.5 pp change is possible just by adapting the categories to the target population. Improvements from the older AlexNet CNN architecture to much more advanced ResNet CNN are very slim when tested on a population re-targeted dataset.

• *Not all dataset biases are created equal.*

Dataset bias has a negative connotation in the field of AI—it suggests poorly curated data. However, this work essentially *introduces* the bias into the dataset, with the aim to bias the dataset *towards* human subjects, who will eventually be likely users of the technology in question. Even more, as the users may come from different sociological and cultural backgrounds, the dataset *should* be biased specifically with the target population in mind.

• *User testing in the evaluation of CNNs is needed.*

The qualitative analysis has shown some surprises that were not expected. The appearance of ‘exotic’ food categories in *Chinese food* after adapting the dataset to *European users* is a strong signal that only user tests can provide insight into what people expect from an AI-powered appliance. The findings of the research can be interpreted as: European users would like to be alerted to the food that they do not usually consume, and this is their serious concern when recognizing food.

• *In many cases, mistakes, made by CNNs are grave.*

The qualitative analysis also revealed that mistakes made by CNNs are grave in the sense that are below what humans expect from state-of-the-art technology (e.g., mixing fish and birds in ImageNet test, mixing meat and sea cucumbers in the food recognition test). Figure 8, Figure 14 and Figure 15 show the natural consequence of CNNs relying only on low-level visual features, and still being unable to infer high-level concepts.

The research, documented in this article, presents a complete methodology that can be employed by anyone seeking to develop or deploy a *user-friendly* AI device or algorithm where the target-user of the system is a human.

The main disadvantage of the proposed method is using online user-studies as they cannot be fully controlled. Online user-studies produce many outliers as humans are not taking the experiment seriously enough. Furthermore, the cost of the user-studies can be prohibitive if statistical tests output a large number of subjects needed for the evaluation. As the datasets are getting larger by the day the method’s statistical power could become irrelevant. Next, the method uses the manual method to determine the optimal number of clusters. In this regard, it cannot be fully automated. Finally, there are multiple parameters that must be considered when designing tests for comparing humans to a machine.

Still, there is room for improvement. To overcome problems that are inevitably brought by using online experiments, one could use offline experiments for better control or more sophisticated web platforms that would automatically detect if humans are taking the experiment seriously.

Next, a better method to determine the number of clusters could be used, for example, adding the means to automatically seek a change of slope in a graph. The different cost functions for categorical data could be used in the clustering process, possibly reducing the number of clusters that got the same label in the labeling task. Using fuzzy set-based k-Modes algorithms [60] could also be considered.

The definition of a labeling task as an open-world problem poses certain challenges. People can label the image as anything, and it is down to a spell checker that will try to correct miss-spelled words. The online correction and suggestion tool would probably dramatically reduce the number of spelling errors and would give users a chance to benefit from the online spelling dictionary. Furthermore, different noise handling techniques for crowd-sourcing could be used [61].

Furthermore, there is still a question what will happen if CNN is trained on labels already adapted to the target user population? Similar to semantic segmentation, it could be only fine-tuned (only the top-most classification layers re-trained). The process is more time-consuming than the approach presented in this work, but it could bring improvements to the methodology.

What this work opens up is a new methodology to examine the differences between a human and a machine in object recognition tasks. With the preposition, the output of already pre-trained object classification algorithms is re-targeted to the user-friendly categorization. The methodology could also be efficiently used the other way around. A CNN pre-trained on an arbitrary dataset could be checked to which population the dataset fits the most and then used for that population. This is similar to transfer learning, except a CNN is transferred to the best target user population.

## Figures and Tables

**Table 1:** ILSVRC2012 categories, selected to form smaller dataset $D^{\prime }$. <web site> and <library> are assumed to be easy for people to recognize, the rest are expected to be hard. Descriptions provided by WordNet [47].

Category	WordNet Description
<site>	A computer connected to the internet that maintains a series of web pages on the World Wide Web.
<library>	A building that houses a collection of books and other materials.
<dunlin>	Small common sandpiper that breeds in northern or Arctic regions and winters in southern United States or Mediterranean regions
<bolete>	Any fungus of the family Boletaceae
<jacamar>	Tropical American insectivorous bird having a long sharp bill and iridescent green or bronze plumage
<gyromitra>	Any fungus of the genus Gyromitra
<dhole>	Fierce wild dog of the forests of central and southeast Asia that hunts in packs
<kakatoe galerita>	White cockatoo with a yellow erectile crest
<earthstar>	Any fungus of the family Geastraceae; in form suggesting a puffball whose outer peridium splits into the shape of a star.
<siamang>	Large black gibbon of Sumatra having the 2nd and 3rd toes partially united by a web.

**Table 2:** VireoFood-172 meat categories, randomly selected to form smaller dataset $D^{\prime }$.

No.	Category
1.	<pickles, shredded pork & vermicelli>
2.	<fried lamb with cumin>
3.	<four-joy meatballs>
4.	<sauteed bullfrog with pickled peppers>
5.	<saute spicy chicken>
6.	<spare ribs with garlic>
7.	<chicken feet with pickled peppers>
8.	<sauteed shredded pork with skin of tofu>
9.	<braised beef with brown sauce>
10.	<sauteed shredded pork with garlic sprout>
11.	<roast chicken wings>
12.	<braised intestines in brown sauce>
13.	<braised pork>
14.	<sauteed snails>
15.	<beefsteak>
16.	<pork with garlic sauce>

**Table 3:** Transformation of the dataset $D^{\prime }$ labels into the cognitive relevant labels.

No.	Transformed Category	Original Categories
1.	<bird>	<dunlin>, <jacamar>, <kakatoe galerita>
2.	<book>	<library>
3.	<fox>	<dhole>
4.	<library>	<library>
5.	<unknown>	<library>, <bolete>
6.	<monkey>	<siamang>
7.	<mushroom>	<bolete>, <gyromitra>, <earthstar>
8.	<website>	<site>

**Table 4:** Metrics of human subjects and the selected deep neural networks AlexNet [55], VGG19 [56], and ResNet152v2 [57] on the pre-CRT (original) and post-CRT (changed) ILSVRC2012 subset $D^{\prime }$.

Model	Top-1 ACC (%)		Precision (%)		Recall (%)		F1-Score (%)	
	Pre	Post	Pre	Post	Pre	Post	Pre	Post
Human population	99.38	99.38	90.91	96.88	90.34	99.74	90.62	98.08
AlexNet	88.13	88.13	90.91	88.13	80.11	84.38	84.66	79.23
ResNet152v2	96.88	96.88	90.91	89.58	88.07	95.57	89.42	88.74
VGG19	94.38	94.38	90.91	88.75	85.8	91.92	88.08	85.26

**Table 5:** Transformation of subset $D^{\prime }$ labels into transformed labels for ENG demography.

Original Categories	Transformed Category							
	<meat>	<meatball>	<chicken foot>	<noodle>	<stew>	<snail>	<vegetable>	<fry stir>
<sauteed shredded pork with garlic sprout>	✔						✔	✔
<braised intestines in brown sauce>	✔						✔	
<fried lamb with cumin>	✔							✔
<spare ribs with garlic>	✔						✔	
<roast chicken wings>	✔							
<beefsteak>	✔							
<braised pork>	✔							
<braised beef with brown sauce>	✔						✔	✔
<sauteed bullfrog with pickled peppers>		✔	✔	✔	✔			
<pork with garlic sauce>		✔	✔	✔	✔			
<four-joy meatballs>		✔				✔		
<sauteed shredded pork with skin of tofu>		✔		✔				
<pickles, shredded pork & vermicelli>		✔	✔	✔				
<saute spicy chicken>		✔	✔		✔			
<chicken feet with pickled peppers>			✔					
<sauteed snails>			✔	✔	✔	✔		

**Table 6:** Transformation of subset $D^{\prime }$ labels into transformed labels for ASIA demography.

Original Categories	Transformed Category						
	<pork>	<meat>	<meatball>	<seafood>	<intestine>	<noodle>	<vegetable>
<braised intestines in brown sauce>	✔	✔			✔		
<fried lamb with cumin>	✔	✔				✔	✔
<spare ribs with garlic>	✔	✔					
<roast chicken wings>	✔	✔					
<beefsteak>	✔	✔				✔	
<braised pork>	✔	✔					
<braised beef with brown sauce>	✔	✔				✔	
<sauteed bullfrog with pickled peppers>		✔	✔	✔			
<pork with garlic sauce>		✔	✔	✔		✔	
<four-joy meatballs>		✔	✔	✔			
<chicken feet with pickled peppers>		✔	✔	✔			
<saute spicy chicken>		✔	✔	✔		✔	
<sauteed snails>			✔	✔		✔	
<pickles, shredded pork & vermicelli>				✔		✔	
<sauteed shredded pork with skin of tofu>						✔	
<sauteed shredded pork with garlic sprout>							✔

**Table 7:** Metrics of ENG human subjects and the selected deep neural networks AlexNet [55], VGG19 [56], and ResNet152v2 [57] on the pre-CRT (original) and post-CRT (changed) ILSVRC2012 subset $D^{\prime }$.

Model	Top-1 ACC (%)		Precision (%)		Recall (%)		F1-Score (%)	
	**Pre**	**Post**	**Pre**	**Post**	**Pre**	**Post**	**Pre**	**Post**
AlexNet	89.93	90.63	93.11	88.79	84.64	81.4	88.42	84.86
Human	100	100	100	100	100	100	100	100
ResNet152v2	93.4	94.1	93.42	88.89	87.91	84.72	90.42	86.72
VGG19	93.4	94.44	93.07	88.89	87.91	83.9	90.33	86.27

**Table 8:** Metrics of ASIA human subjects and the selected deep neural networks AlexNet [55], VGG19 [56], and ResNet152v2 [57] on the pre-CRT (original) and post-CRT (changed) ILSVRC2012 subset $D^{\prime }$.

Model	Top-1 ACC (%)		Precision (%)		Recall (%)		F1-Score (%)	
	**Pre**	**Post**	**Pre**	**Post**	**Pre**	**Post**	**Pre**	**Post**
AlexNet	89.93	90.63	93.11	87.21	84.64	80.23	88.42	83.54
Human	100	100	100	100	100	100	100	100
ResNet152v2	93.4	94.1	93.42	87.5	87.91	83.47	90.42	85.41
VGG19	93.4	94.44	93.07	87.5	87.91	83.61	90.33	85.49

## Dataset

Research data related to this submission will be made available on request.

## Abbreviations

The following abbreviations are used in this manuscript:

AI	artificial intelligence
CNN	Convolutional Neural Network
HMO	hierarchical neural network model
ID	identification number
ILSVRC2012	ImageNet Large Scale Visual Recognition Challenge 2012
mAP	mean average precision
ACC	accuracy
CFM	confusion matrix
CRT	cognitive relevance transform
pp	percentage points
ENG	English population
ASIA	Asian population

## Appendix A. ILSVRC2012 Results

Confusion Matrices (CFMs) obtained by the methodology for the ILSVRC2012 dataset are presented here. The results can be easily reproduced for comparative studies. The matrix in Figure A1a is the average confusion matrix for human subjects, directly generated from the human subject classification and Figure A1b is the population confusion matrix, as follows from the transformation by Algorithm 6.

The confusion matrices for CNNs $\mathrm{CFM}(\delta_{M}\left(D\right),D^{\prime })$ were obtained simply by running respective algorithms on the validation set of the selected ILSVRC2012 categories. The worst and best results are shown in Figure A2.

Results after applying CRT to the dataset $D^{\prime }$ are shown in Figure A3a,b for humans and the ResNet, respectively.

## Appendix B. VireoFood-172 Results

Confusion Matrices (CFMs) obtained by the methodology for the VireoFood-172 dataset are presented here. The results can also be easily reproduced for comparative studies. The obtained CFMs for ASIA and ENG subjects and populations are shown in Figure A4 and Figure A5 respectively.

CFMs for AlexNet and ResNet deep neural networks are shown in Figure A6.

Figure A7 shows ENG population and ResNet CFM which were changed by CRT obtained from ENG population.

ResNet CFM was also changed by CRT obtained from the ASIA population. It is shown in Figure A8b alongside ASIA population CFM.
